# P-1155. Acute Osteomyelitis and Septic Arthritis in Children in Israel: Epidemiology, Microbiology, Clinics and Outcomes

**DOI:** 10.1093/ofid/ofae631.1341

**Published:** 2025-01-29

**Authors:** Galia Grisaru, Reut Kassif Lerner, Yael Elmaleh, Nadav Michaan, Gideon Paret

**Affiliations:** Tel Aviv Sourasky Medical Center, Tel Aviv, Tel Aviv, Israel; The Edmond and Lily Safra Children's Hospital, Sheba Medical Center, Tel aviv, Tel Aviv, Israel; Tel Aviv Sourasky Medical Center, Tel Aviv, Tel Aviv, Israel; Tel Aviv Sourasky Medical Center, Tel Aviv, Tel Aviv, Israel; The Edmond and Lily Safra Children's Hospital, Sheba Medical Center, Tel Hashomer, Ramat Gan, Tel Aviv, Israel

## Abstract

**Background:**

The goal of this study was to characterize the clinical, microbiologic epidemiological characteristics and outcome associated with osteoarticular infections ( OAIs) in hospitalized children in Israel.

**Figure 1:**
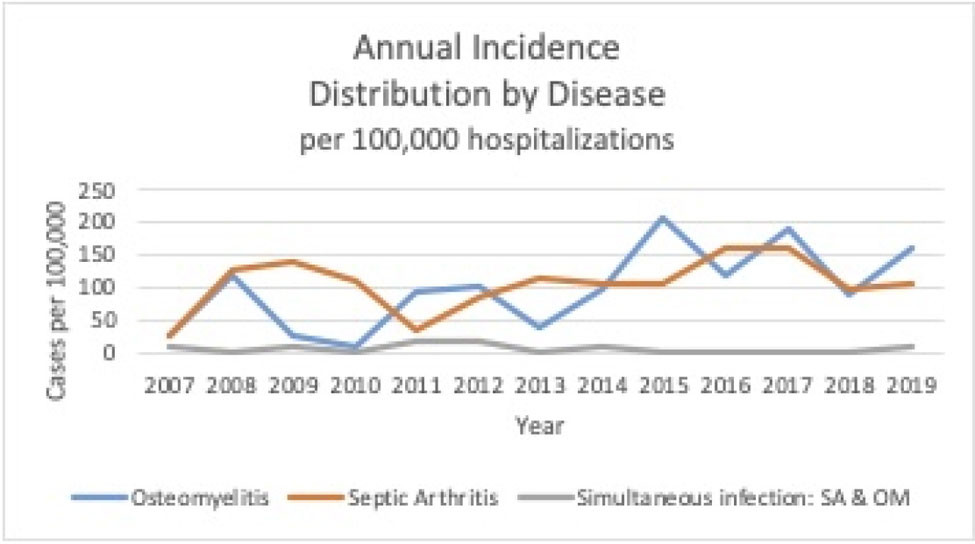
Annual incidence for each disease per 100,000 hospitalizations during the years of study. SA-septic arthritis, OM-osteomyelitis

**Methods:**

Patients < 18 years old admitted with a diagnosis of acute osteomyelitis (AOM) or septic arthritis ( SA), between January 2007 and December 2019 were included if they had a microbiological and/or radiological findings supporting the diagnosis. Clinical and laboratory data were retrieved from the medical records.

**Figure:**
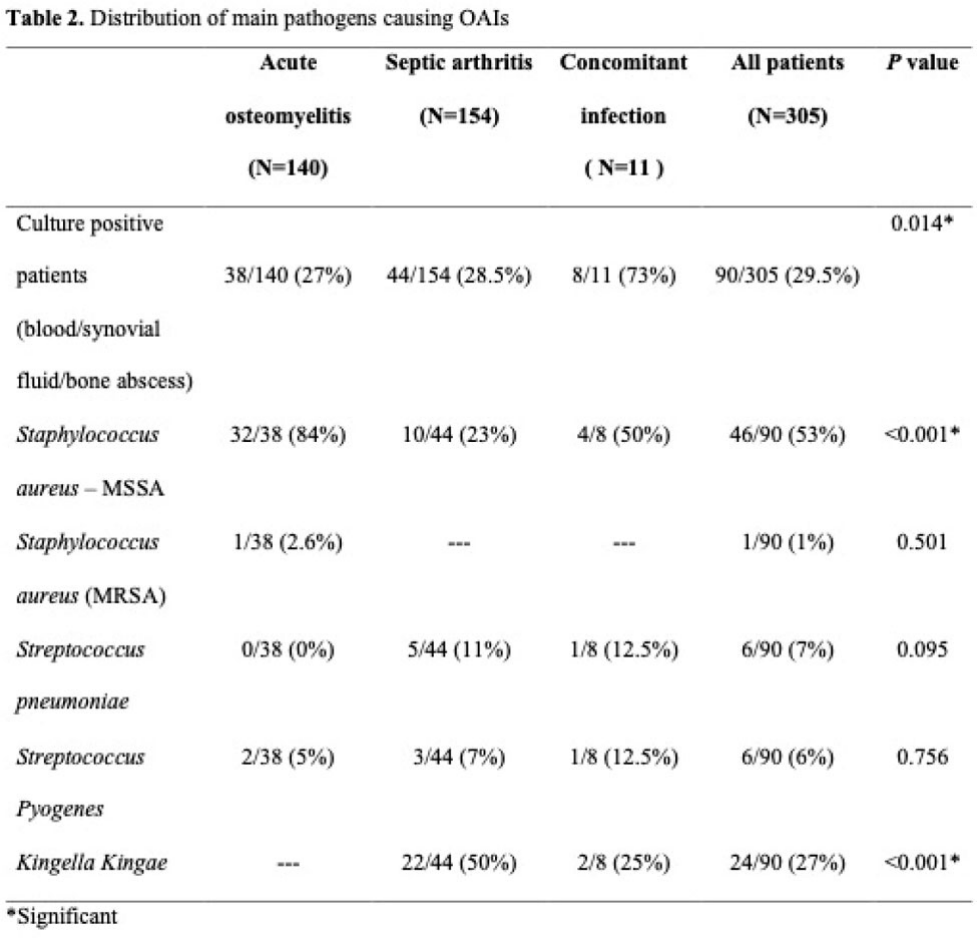

**Results:**

305 children with OAIs were included. The annual incidence among osteomyelitis patients increased by 10.25 cases per 100,000 hospitalizations every year (*P*=0.019) during the study period. Tibia and knee were the most frequently involved bones and joints. About one-half (47.5%) of the patients were febrile upon admission. Laboratory data analyses showed that 36 (26%) AOM and 74 (48%) SA patients had leukocytosis at presentation. C-reactive protein (CRP) levels ( >5 mg\dL) were elevated in 87% and 71% of patients with SA, and AOM, respectively. The most common isolated pathogens were methicillin susceptible *Staphylococcus aureus* followed by *Kingella Kingae*. About 5% of the hospitalized patients due to septic arthritis and 6% of the osteomyelitis patients had complications and underwent a surgical intervention. There were no long term sequelae.

**Figure:**
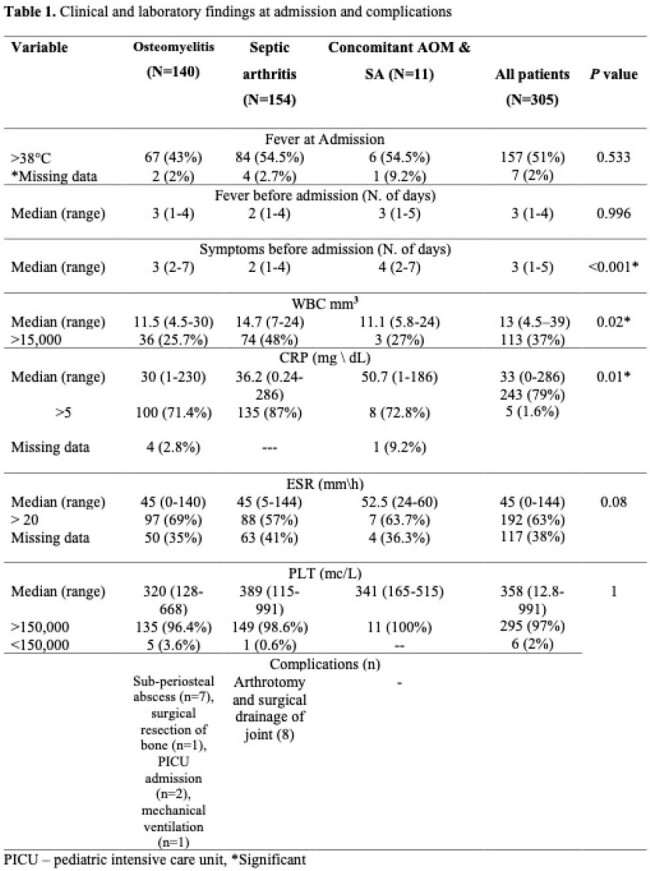

**Conclusion:**

The admission rate for acute osteomyelitis in children increased significantly during the last decade. Fever and WBC, were not sufficiently reliable to diagnose or rule out OAIs. The most common isolated pathogens are methicillin susceptible *Staphylococcus aureus* followed by *Kingella Kingae*. Through accurate diagnosis and treatment with appropriate antibiotics and surgical intervention when indicated osteoarticular infections have a favorable outcome and no long term sequelae.

**Disclosures:**

**All Authors**: No reported disclosures

